# Didactic Benefits of Surgery on Body Donors during Live Surgery Events in Minimally Invasive Surgery

**DOI:** 10.3390/jcm9092912

**Published:** 2020-09-09

**Authors:** Johannes Ackermann, Thilo Wedel, Bernd Holthaus, Bernd Bojahr, Andreas Hackethal, Sara Brucker, Matthias Biebl, Martina Westermann, Veronika Günther, Magret Krüger, Nicolai Maass, Liselotte Mettler, Göntje Peters, Ibrahim Alkatout

**Affiliations:** 1 Department of Obstetrics and Gynecology, Kiel School of Gynaecological Endoscopy, University Hospitals Schleswig-Holstein, Campus Kiel, Arnold-Heller Str. 3, House C, 24105 Kiel, Germany; johannes.ackermann@uksh.de (J.A.); martina-b.baier@web.de (M.W.); veronika.guenther@uksh.de (V.G.); magret.krueger@uksh.de (M.K.); nicolai.maass@uksh.de (N.M.); profmettler@gmx.de (L.M.); goentje.peters@uksh.de (G.P.); 2 Institute of Anatomy, Christian-Albrechts University Kiel, Otto-Hahn-Platz 8, 24118 Kiel, Germany; t.wedel@anat.uni-kiel.de; 3 Clinic of Obstetrics and Gynecology, St. Elisabeth Hospital, 49401 Damme, Germany; b.holthaus@t-online.de; 4 Clinic of Minimally Invasive Surgery, Kurstraße 11, 14129 Berlin-Zehlendorf, Germany; b.bojahr@mic-berlin.de; 5 Frauenklinik an der Elbe, Oberbaumbrücke 1, 20457 Hamburg, Germany; andreashackethal@googlemail.com; 6 Department für Frauengesundheit, University Hospital Tübingen, Calwer Straße 7, 72076 Tübingen, Germany; sara.brucker@med.uni-tuebingen.de; 7 Department of Surgery, Campus Virchow Klinikum, Charité - Universitätsmedizin Berlin, Augustenburger Platz 1, 13353 Berlin, Germany; matthias.biebl@charite.de

**Keywords:** body donors, laparoscopy, minimally invasive surgery, surgical education, clinical anatomy, live surgery events

## Abstract

Background: Live surgery events serve as a valuable tool for surgical education, but also raise ethical concerns about patient safety and professional performance. In the present study, we evaluate the technical feasibility and didactic benefits of live surgery on body donors compared to real patients. Methods: A live surgery session performed on a body donor’s cadaver embalmed in ethanol–glycerol–lysoformin was integrated into the live surgery program presented at a major gynecological convention of minimally invasive surgery. Surgical procedures carried out in real patients were paralleled in the body donor, including the dissection and illustration of surgically relevant anatomical landmarks. A standardized questionnaire was filled by the participants (*n* = 208) to evaluate the appropriateness, effectiveness, and benefits of this novel concept. Results: The live surgery event was appreciated as a useful educational tool. With regard to the use of body donors, authenticity was rated high (85.5%), and the overall value of body donors for surgical education and training was rated very high (95.0%). The didactic benefit of simultaneous operations performed on body donors and real patients was considered particularly useful (95.5%), whereas complete replacement of real patients by body donors was not favored (14.5%). Conclusions: The study demonstrated both the technical feasibility and didactic benefits of performing minimally invasive surgery in body donors as part of live surgery events. This novel concept has the potential to enhance anatomical knowledge, providing insights into complex surgical procedures, and may serve to overcome yet unresolved ethical concerns related to live surgery events.

## 1. Introduction

Learning gross human anatomy by means of systematic dissection of body donors has always been a fundamental element of medical education [1]. Anatomy is usually taught at the beginning of medical school, in dissection courses on body donors conventionally fixed in formaldehyde solutions [2]. Knowledge of human anatomy is the basis of any medical intervention. However, manipulation within the human body and the refinement of skills are usually achieved on real patients.

In view of the increasingly limited human and financial resources, as well as higher ethical standards in modern medicine, surgical education must necessarily encompass new training concepts [3,4]. Rapid advancements in medical and digital technology, especially minimally invasive surgery, have resulted in a wide range of training and educational opportunities, such as virtual reality training devices or interactive video learning platforms [5,6,7,8]. In view of these new options, the traditional concept of acquiring knowledge of surgical anatomy on vulnerable patients entrusted to our care appears to be debatable, at least from the ethical point of view [9,10].

Surgical training courses using human body donors are becoming increasingly important in curricular and postgraduate education [11,12,13]. Novel fixation techniques have been developed recently in order to meet this increased demand [14]. One of these techniques is ethanol–glycerol–lysoformin fixation, which is relatively simple and cost effective, and provides realistic tissue and organ properties [15]. We established the suitability of this method for minimally invasive surgical procedures, and demonstrated its didactic benefits for the acquisition and refinement of surgical skills [16,17].

Live surgery events serve as a useful additional platform for training and learning surgery as well as clinical anatomy [18]. In fact, live surgery events constitute a core element of surgical conventions [19]. Leading experts in their respective fields demonstrate live surgeries, which frequently include novel surgical techniques and devices applied on real patients [20]. Typically, the attendees are able to communicate with the surgeons during video transmission [18]. A large number of surgeons are introduced to new surgical techniques and the relevant clinical anatomy is demonstrated on a single patient [19,21]. Furthermore, live surgery events offer the opportunity to learn from experts as a role model in real life, as well as handle surgical complications and manage difficult cases appropriately [22,23]. Such events are especially attractive in minimally invasive surgery because the perspective of the operating surgeon is directly transmitted to the attendees in the auditorium, who then participate virtually in the operation [20].

However, live surgery events are controversial because of medical and ethical concerns [19,24]. In fact, live surgery is known to be associated with prolonged operating and anesthesia times, lower rates of therapeutic success, and delayed time to intervention [25,26]. These disadvantages may not be acceptable under the supreme medical ethics of doing no harm [19]. Some professional associations have issued recommendations for the improvement of these concerns and offered congress organizers suitable guidelines to overcome these problems [22,27].

Based on these considerations, in the present study we evaluated a live surgery event supplemented by minimally invasive surgical procedures performed on a body donor, along with practical demonstrations of surgical anatomy. The rationale for implementing this novel module into a conventional live surgery session was to assess the didactic quality and benefits perceived by the attendees, and the potential reduction in risks associated with live surgery on real patients.

## 2. Materials and Methods

### 2.1. Format of the Live Surgery Event

**Figure 1:**
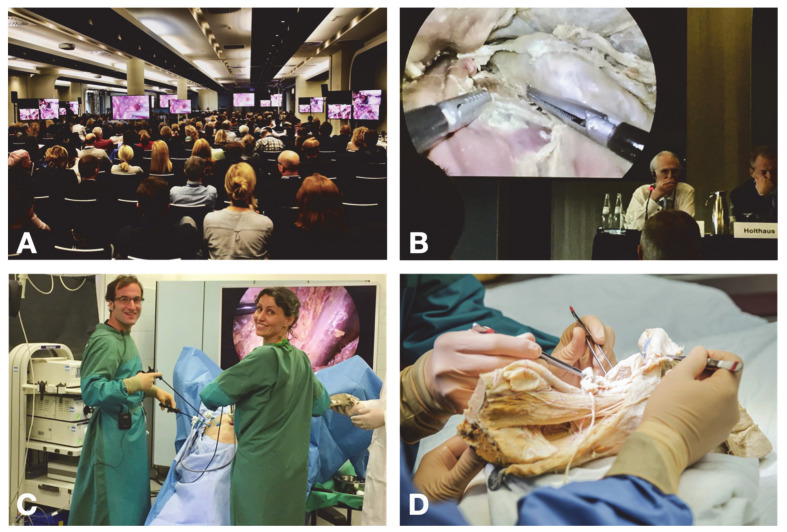
Set-up of the live surgery event. (**A**): Conference room equipped with several HD monitors for transmission of the live surgery event. (**B**): Transmission of laparoscopic procedures performed on a body donor from the attendees’ perspective. (**C**): Technical setting for live surgery performed on a body donor. (**D**): Demonstration of relevant anatomical structures on a formalin-fixed pre-dissected anatomical specimen (hemipelvis).

The format of the 23rd Annual Meeting of the Gynecological Endoscopy Working Group (AGE) (2018, Hamburg/Germany) included a live surgery session on real patients from two hospitals in Hamburg (Agaplesion Diakonieklinikum Hamburg, Frauenklinik an der Elbe) paralleled by laparoscopic operations on a body donor, transmitted from the operating room in the institute of anatomy at Kiel University (Kiel/Germany). All transmissions were carried out by a professional broadcast team (TV-Studio Leonberg, Gerlingen/Germany) and presented on several large-sized HD monitors placed throughout the entire congress hall to allow optimal visibility from all seats (Figure 1A). The surgeries were transmitted simultaneously from the hospitals and the institute of anatomy, but only one source was presented to the auditorium at a time. Communication between surgeons and the auditorium was coordinated by the chairmen (BH, SB, LM, BB, ES, NM), who commented on the surgical procedures and passed questions from the auditorium to the operating surgeons.

### 2.2. Laparoscopy on Real Patients

Patients with benign (deep infiltrating endometriosis, uterine fibroids, genital prolapse) and malignant (endometrial and cervical cancer) gynecological diseases were selected for live laparoscopic surgery. All of the operations were performed by a surgeon from the presenting hospitals in cooperation with an invited faculty surgeon who was given adequate time to study the cases. All patients were informed previously about the specific conditions of the live surgery event, had given their written consent, and could meet their respective surgeons the day before the operation. Participation was absolutely voluntary and devoid of any financial advantage. The operations were performed in accordance with current medical knowledge, by surgeons (AH and others) experienced in live surgery events and with the highest certification levels of the AGE.

### 2.3. Laparoscopy on Body Donor

The body of a female body donor (77 years, 59 kg) was obtained from the body donation program of the institute of anatomy at Kiel University. Prior to her death, the donor had given her written consent to the use of her body for educational and research purposes. Advanced stages of arteriosclerosis and previous abdominal surgery were excluded to allow efficient perfusion fixation and optimal conditions for laparoscopic surgery. Exploratory laparoscopy was performed before the live surgery event to confirm the presence of the uterus and adnexa, and exclude severe adhesions or other major pathologies. The detailed fixation procedure has been reported previously [16]. Briefly, the body donor was perfused with a fixative solution (70% ethanol, 30% glycerin, 0.3% lysoformin) administered at a ratio of 0.3 l/kg body weight via the femoral artery. Perfusion was carried out by alternating cycles of injections (30 min) and breaks (20 min) over a period of about 24 h. The fixed body donor was draped in cloths moistened with a watery solution supplemented with 1% thymol, placed in a sealed plastic bag, and stored at 4 °C until use.

Laparoscopic surgery was performed in an operating room at the institute of anatomy by two experienced surgeons (IA, GP) and accompanied by a clinical anatomist (TW). The body donor was safely mounted on a mobile operating table to allow optimal positioning. The laparoscopic equipment included an endoscopy system, CO_2_ insufflation, a rinsing device, and standard laparoscopic instruments (Figure 1B,C). The aims of laparoscopic procedures carried out on the body donor were twofold. The first of these was that the key steps of live surgery performed on real patients were to be paralleled on the body donor, but with more time taken to focus on anatomical structures and landmarks related to the surgical procedures. Moreover, alternative surgical approaches and modified techniques were demonstrated; for obvious reasons, these could not be shown in the live surgery sessions. Second, special emphasis was given to the dissection of those anatomical regions with structures exposed to the risk of injury, such as the autonomic nerve plexus in the para-aortic and presacral region, the obturator nerve in the obturator fossa, the genitofemoral nerve passing along the psoas muscle and external iliac vessels towards the groin region, or the course of the ureter from the pararectal region throughout the parametrial space towards the bladder. In addition, anatomical structures rarely seen during conventional laparoscopic procedures were explicitly exposed and discussed, such as the ventral roots of the spinal nerves L5-S4, the retrorectal space, branches of the posterior division of the internal iliac vessels, and lumbar vessels.

### 2.4. Demonstration of Pre-Dissected Anatomical Specimens

The same team (TW, IA, GP) demonstrated selected pre-dissected formalin-fixed specimens to highlight those anatomical structures which could not be entirely dissected during the laparoscopic procedures, but were considered relevant for live surgeries (Figure 1D). The interactive demonstration included the pelvic fascial system, pelvic floor muscles and ligaments, the pelvic and para-aortic lymphatic drainage system, and the inferior hypogastric plexus with terminal branches.

### 2.5. Evaluation

After the live surgery event, all participants were invited to evaluate the session on a questionnaire (Table 1). The evaluation focused on two major aspects: (1) the benefits of live surgery events for surgical education, prevention of complications, learning new surgical techniques, and improving personal surgical skills; (2) the value of live surgery on a body donor for surgical training, the authenticity of the body donor, the benefit of simultaneous surgery on real patients and body donors, and the potential for body donors to replace real patients at live surgery events.

The questionnaire was approved by a statistician and a medical ethics specialist. The answers were recorded on a continuous visual analog scale (VAS) and expressed in percentages (0: very low; 100: very high). The questionnaire recorded age, gender, professional qualification, type of medical care institution, AGE membership, the level (MIC I–III) of skills in minimally invasive surgery according to the AGE criteria (certification criteria are listed on the AGE website [28]), and the number of live surgery events attended in the past. Finally, free optional text fields were provided for appreciation and criticism. The study was approved by the ethics committee of the Medical Faculty of Kiel University (approval number D 453/18).

### 2.6. Statistical Analysis

The IBM SPSS Statistics 23 program was used for statistical analysis. Quantitative variables were presented descriptively as means and standard deviations, minimum, maximum, quartiles and interquartile ranges (IQR), and tested for normality with the Kolmogorov–Smirnov test. VAS scores were assessed as follows: <20, very low; 20 to <40, low; 40 to <60, moderate; 60 to <80, high; 80 to 100, very high. A correlation analysis was performed to determine the influence of age and the number of live surgery events attended in the past. When significant deviations from normal distribution were found, we used the Spearman-rho test for correlation analysis. The correlation coefficient (r) was evaluated as follows: r ≤ 0.2, no correlation; 0.2 < r ≤ 0.5, weak to moderate correlation; 0.5 < r ≤ 0.8, strong correlation; 0.8 < r ≤ 1.0, very strong correlation. Tests were performed bilaterally and the level of significance was set to 5% (*p* < 0.05). The Mann–Whitney U-test was used for subgroup analysis of nonparametric data, or the Kruskal–Wallis test for more than two subgroups. Tests were performed bilaterally and the level of significance was set to 5% (*p* < 0.05).

## 3. Results

### 3.1. Demographic Data

A total of 487 participants had registered for the live-surgery event day at the AGE congress. 208 participants (50.5% female, 47.6% male) completed the questionnaire after the live surgery session. As the exact number of participants who attended the live surgery event was not recorded, the return rate could not be determined. Assuming that all registered attendees participated in the live surgery session, the response rate would be 42.7%. The majority of the participants were members of the AGE (86%), and nearly a half of them (49%) had an MIC II or MIC III certificate. Most participants were specialists in obstetrics and gynecology (90%). Of these, 40 (19.2%) were clinical directors and 44 were senior consultants (21.2%). Most physicians worked in primary and secondary care medical institutions (41%), followed by quaternary (26%) and tertiary care units (25%). The participants had attended an average number of eight live surgery events in the past (Table 2).

### 3.2. Value of Live Surgery Events Performed on Real Patients

**Figure 2:**
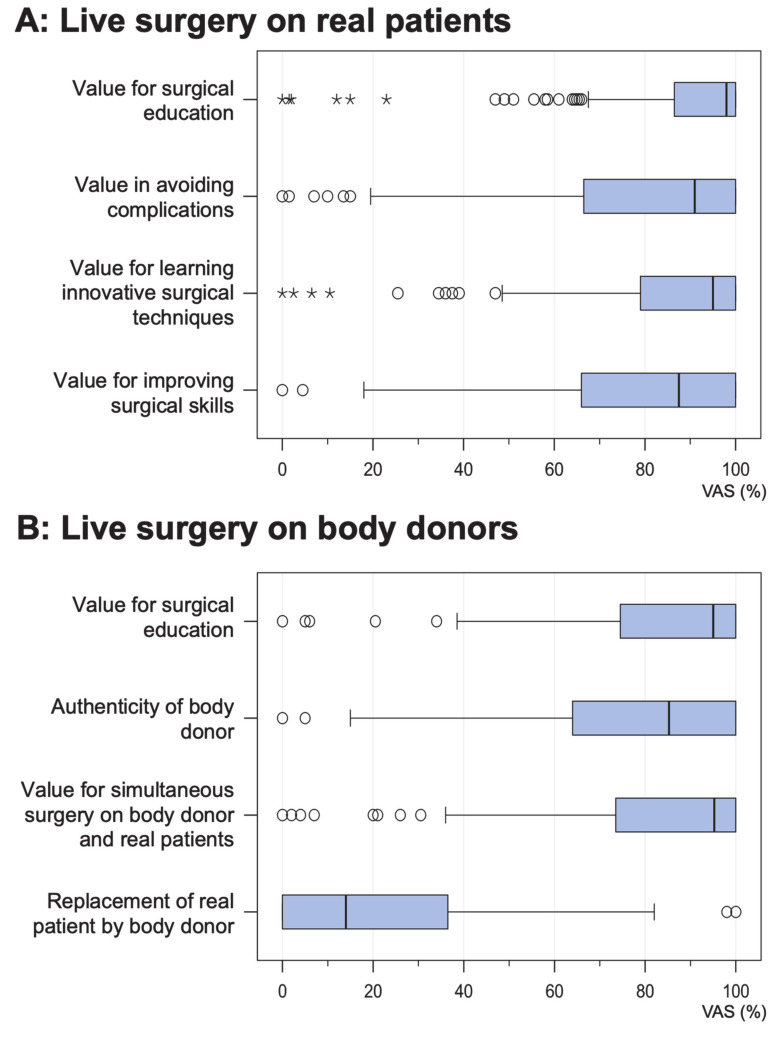
Evaluation (boxplots) of live surgery performed on (**A**) real patients and (**B**) a body donor. The answers were recorded on a continuous visual analog scale (VAS).

The value of live surgery events for surgical education and training was rated “very high” by most participants (median 98.0%, IQR 86.5–100%, *n* = 206). A similar high rating was given to the acquisition of innovative surgical techniques (median 95.0%, IQR 79.0–100%, *n* = 206). When asked to rate the benefits of avoiding complications in their own patients, the attendees’ responses ranged from “very low” to “very high”, but most attendees rated the benefits “very high” (median 91.0%, IQR 66.5–100%, *n* = 205). Finally, the value of improving their own surgical skills was rated “very high” by most participants (median 95.0%, IQR 65.0–100%, *n* = 206). The results are shown in Figure 2A and Table 1.

### 3.3. Value of Live Surgery Events Performed on Body Donors

The value of live surgery events for surgical education and training was rated “very high” by most participants (median 95.0%, IQR 75.0–100%, *n* = 204). Similar ratings were given when the attendees were asked about the value of simultaneous live surgery performed on real patients and body donors (median 95.5%, IQR 74.5–100%, *n* = 206). Most attendees rated the authenticity of the body donors as “high” or “very high” (median 85.5%, IQR 65.5–100%, *n* = 202). In contrast, the option of replacing real patients with body donors at live surgery events was rated “very low” by most participants (median 14.5%, IQR 0–39.0%, *n* = 202). The results are shown in Figure 2B and Table 1.

### 3.4. Integration of Body Donors and Pre-Dissected Anatomical Specimens into Live Surgery Events

The overall feedback of the attendees in the form of free-text comments was very positive. Both the demonstration of key surgical steps in the body donor related to the live surgeries and the illustration of relevant anatomical structures in pre-dissected specimens were highly appreciated. The didactic benefit was confirmed by repeated suggestions to spend more time on demonstrations of anatomical landmarks, and to switch more frequently between the real patient and the body donor during the live transmission. A representative selection of comments is shown in Table 3.

### 3.5. Subgroup Analysis of the Evaluation

**Figure 3:**
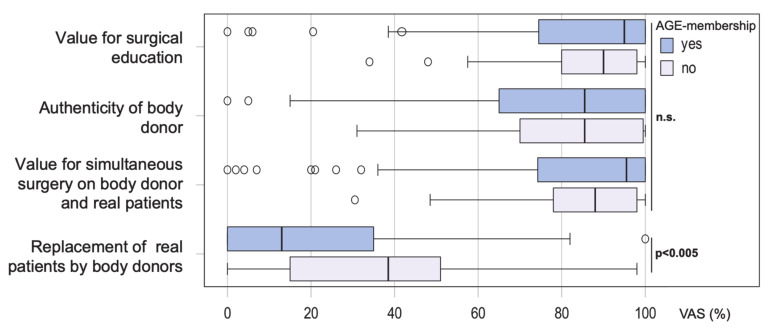
Value of live surgery performed on body donors: comparative subgroup analysis of AGE members and non-members. The answers were recorded on a continuous visual analog scale (VAS).

Subgroup analyses were performed to assess whether the responses depended on specific characteristics of the participants. However, neither age, gender, professional qualification, type of medical care facility, MIC levels, nor the number of previously attended live surgery events had a significant influence on the response pattern. The only significant difference was registered with regard to AGE membership: when asked about the potential of body donors to replace real patients at live surgery events, the approval rate was significantly higher among non-members (*p* < 0.005) (median 38.5%, IQR 13.5–54.5%) than among members (median 13%, IQR 0–35%) (Figure 3).

## 4. Discussion

Knowledge of clinical anatomy is the basis of successful surgery and the acquisition and development of new surgical techniques [29,30,31,32]. The attendance of live surgery events is an established means of learning anatomy as well as novel surgical techniques in nearly all surgical disciplines [20]. However, the demonstration of surgical procedures in real patients at live surgery events is subject to critical discussion from an ethical point of view [21,33]. Several studies have shown a potentially negative impact on the outcome of patients who have undergone live surgery, which is opposed to the no-harm principle of medicine [24,25,26,34]. These critical issues may be overcome by the use of body donors at live surgery events for the illustration of surgical techniques as well as the demonstration of related anatomical features.

Body donors embalmed in ethanol–glycerol–lysoformin were shown to be particularly suitable for surgical training and education [15,16,17,35]. Based on these previous encouraging experiences, laparoscopy performed on body donors was integrated into the live surgery session at the largest German surgical working group in gynecology (AGE) in order to evaluate the authenticity of this educational approach, its value for surgical training, and its didactic benefits. Analysis of the feedback showed that each of these aspects was highly appreciated by the participants. However, most attendees could not conceive the complete replacement of live surgery on real patients by body donor surgery. To the best of our knowledge, the current study is the first to address the concept of integrating surgical procedures on body donors into live surgery events.

The benefit of live surgery events for surgical training has been repeatedly and critically discussed [21,23,36]. However, we observed a high degree of approval for this training concept. The benefits in regard to the three primary training goals (avoidance of complications, innovative gain, improvement in own skills) was especially emphasized by the participants, and is most likely generated by the unique concept of live surgery [19]. The presentation of surgical procedures and complex situations by experts makes their knowledge and skills available to a large audience [19,21,37], and the techniques can be widely adopted in daily patient care [20]. Therefore, the live surgery event is likely to exert a sustainable effect on the quality of surgery for the attendees as well as their patients.

These undoubted advantages are counteracted by the potential health risks experienced by individual patients undergoing surgery at live surgery events [23]. Although live surgery has not been associated with additional risks due to a potentially higher complication rate, we do have evidence of health risks due to prolonged surgery and anesthesia time, a potential delay of treatment, and sometimes lower success rates of treatment [21,24,25,26,34]. These observations are supported by an anonymous assessment of guest surgeons at live surgery events, who questioned the indication for surgery or would even have chosen a different surgical procedure in one half of the cases [38]. Notably, the majority of surgeons involved in the survey would not make themselves available for live surgery as a patient and reported a high level of anxiety in performing a surgical procedure as a guest surgeon in a foreign clinic. On the other hand, the expectations of the audience may induce a greater willingness on the part of the surgeon to take more risks during the transmitted surgical procedure [38]. Moreover, informing the patient correctly about the risks associated with live surgery is a critical issue and will have to be addressed in the future [39]. These aspects illustrate the conflict between medical benefits for general public health and the potential harm to the individual patient arising from live surgery events [22]. However, we lack extensive data on the complications and risks of live surgery events [21]. Further studies will be needed to evaluate pending issues in the interest of patients, as well as provide solutions to the ethical dilemma.

One step towards solving this problem could be the integration of surgical interventions performed on body donors into live surgery events. The additional educational benefit of this concept was clearly revealed in the present study. Simultaneous surgery performed on real patients and body donors was especially appreciated by the participants. The quality and authenticity of the body donors were also considered very realistic, thus confirming previous data about the suitability of ethanol–glycerol–lysoformin fixation for this purpose [16,17]. Moreover, the use of body donors makes it possible to demonstrate the patient’s relevant anatomy beyond the limits observed in real patients. Thus, topographic relationships between the susceptible anatomical structures can be displayed and explained, and will help to avoid or handle complications encountered in real patients. The attendees’ enthusiasm for the additional option of anatomical demonstrations supports the didactic value of this concept.

The combined approach may reduce the time of surgery and anesthesia in live surgery, as all anatomical issues, alternative surgical procedures and technical variations can be presented without stress and time pressure in body donors. In addition, the combined use of both body donors and real patients allows one to focus on a broader spectrum of surgical procedures, and at the same time will reduce the number of live surgeries, as several interventions can also be illustrated in body donors. Finally, the synergistic effects of both approaches may optimize the use of existing resources and enhance appreciation of the patient’s willingness to make himself/herself available for medical training.

The question arises as to whether the real-patient scenario at live surgery events can be replaced completely by body donors. Our survey showed a clearly defensive attitude on the part of most participants, suggesting that a complete renunciation of the real patient might cause an unacceptable loss of educational and sustainable quality. However, the survey refers to a special setting, focused exclusively on laparoscopic gynecological operations. The statement may not be directly transferrable to other surgical disciplines. Furthermore, selected operations could possibly be performed equally well on body donors and real patients. We conclude that the primary value of surgical interventions on body donors is that it complements traditional live surgery by providing additional anatomical training concepts in terms of “where do I operate?”, whereas live surgery on real patients is intended to teach surgical steps and techniques in terms of “how do I operate?” In this respect, the different evaluation of AGE members and non-members is an interesting aspect. While the negative attitude of AGE members was more pronounced than that of non-members, no difference was registered with regard to other sociodemographic factors. The lower level of habituation and adherence to traditions among non-members could be one explanation for this phenomenon.

In summary, the study demonstrated the technical feasibility and educational potential of surgical interventions performed on body donors at live surgery events in minimally invasive surgery. The feedback of the participants proves that the demonstration of surgically relevant anatomical landmarks as permitted by the use of body donors was of considerable benefit in clinical routine. The attendees’ positive appraisal favors the integration of this concept as a complementary module in live surgery events, and could potentially resolve the associated ethical concerns. We hope that this “proof-of-principle” may contribute to future discussions concerning the modification of live surgery events in terms of combining surgery on real patients with interventions on body donors.

## Figures and Tables

**Table 1:** Questionnaire: items and results.

	N	Mean (SD)%	Min./ Max.	Median (IQR)%
1. How do you rate the benefit of live surgery on real patients for surgical training and further education?	206	88.6 ± 19.7	0/100	98.0 (86.5–100)
2. How do you rate the benefit of live surgery on real patients to avoid complications in your own patients?	205	79.6 ± 25.5	0/100	95.0 (79.0–100)
3. How do you rate the benefit of live surgery on real patients for learning innovative surgical techniques?	206	85.6 ± 20.7	0/100	91.0 (66.5–100)
4. How do you rate the benefit of live surgery on real patients for improving your own surgical skills?	206	79.1 ± 24.0	0/100	87.5 (65.0–100)
5. How do you rate the benefit of live surgery on the body donor for surgical training and further education?	204	84.4 ± 21.2	0/100	95.0 (75.0–100)
6. How do you rate the authenticity of the body donor?	202	78.9 ± 22.6	0/100	85.5 (65.5–100)
7. How do you rate the educational value of simultaneous surgery on body donors and real patients?	206	82.8 ± 24.2	0/100	95.5 (74.5–100)
8. Could the body donor replace the real patient in live surgery events?	202	23.3 ± 25.7	0/100	14.5 (0–39.0)

**Table 2:** Description of participants.

Total Number	208 (100%)
Age (median)	45 years (range, 25–78 years)
Number of previously attended live surgery events (median)	8 (range: 0–100)
Gender	
Female	105 (50.5%)
Male	99 (47.6%)
AGE membership	
Yes	178 (86%)
No	25 (14%)
AGE certification	
MIC I	57 (28.2%)
MIC II	83 (41.1%)
MIC III	16 (7.9%)
no certification	47 (22.6%)
Professional experience	
Resident	11 (5.3%)
Specialist	24 (11.5%)
Consultant	81 (38.9%)
Senior consultant	44 (21.2%)
Clinical director	40 (19.2%)
Medical care unit	
Primary and secondary care	82 (41.0%)
Tertiary care	50 (25.0%)
Quaternary care	52 (26.0%)
Private medical office with a surgical unit	15 (7.5%)

**Table 3:** Praise and criticism of live surgery performed on body donors.

What Did You Like?	What Did You Not Like?	What Should Be Done Differently?
“Parallel surgical steps on body donor and patient.” “Detailed presentation and explanation of the anatomical structures.” “Simultaneous laparoscopy on both real patients and the body donor during the live surgery session was the highlight of the congress.”	“Too little time allocated to anatomical dissection and laparoscopy on the body donor.” “The start of the anatomical presentation was too early, as many participants were not present yet.” “Suboptimal transmission on video screens.”	“More transmissions from the anatomy operating room.” “More time to combine anatomical demonstration with live surgery.” “The videos, especially from the anatomy lab, should be made available to the participants.” “Better scheduling of the anatomy block, so that more aspects can be shown.” “Switch more frequently between live surgery and the anatomy lab.”
